# Microstructure and chemorheological behavior of whipped cream as affected by rice bran protein addition

**DOI:** 10.1002/fsn3.939

**Published:** 2019-01-28

**Authors:** Azade Ghorbani‐HasanSaraei, Ali Rafe, Seyed‐Ahmad Shahidi, Azin Atashzar

**Affiliations:** ^1^ Department of Food Science and Technology College of Agriculture and Food Science Ayatollah Amoli Branch Islamic Azad University Amol Iran; ^2^ Department of Food Processing Research Institute of Food Science and Technology (RIFST) Mashhad Iran

**Keywords:** dairy, microstructure, rheology, rice bran protein, stability, whipping cream

## Abstract

The effect of rice bran protein (RBP) isolate addition on the rheological and structural properties of commercial whipped cream with 25% and 35% fat was investigated. Results showed that increasing the fat content from 25% to 35% leads to an increase in the elastic modulus. Furthermore, by increasing the amount of RBP from 1% to 3% in both creams, significant increase occurred in the complex modulus. As the fat content increased from 25% to 35%, the slope of flow behavior was increased, which revealed more thinning behavior and pseudoplasticity index of cream. The cream containing 35% fat and 3% RBP had also shown the low index (*n* = 0.298) which confirmed the firmer structure of the cream. The maximum consistency index (*k*) obtained was 9.41 for the cream with 35% fat and 3% RBP, which approved its strong foam structure. In general, according to our results it is obvious that whipped cream with the highest amount of fat and the lowest value of protein can lead to maximum stability of the whipping cream. Among the samples, the lowest stiffness was observed in cream of 35% fat, containing 3% rice bran protein. However, cream containing 35% fat and 1% RBP had convenient overrun and good stability. The microstructural results showed that the cream structure has relatively large globular aggregates in network and develops large pores, which permit to retain sufficient water/air. By increasing the fat content of cream from 25% to 35%, the voids and spaces in the cream were significantly decreased and the pores become less which improve the foam structure. Therefore, it can be concluded the cream with more fat has the more overrun and stability. In general, it is possible to improve the foam structure of cream by substituting fat by RBP.

## 1 INTRODUCTION

Whipped cream is one of the most delicious dairy products used in dessert, pastries, cakes, and ice cream. It is a complex foam structure in which partially coalesced fat droplets stabilize air bubbles at the air–water interface. Air bubble introduction into whipping cream by mechanical agitation forms a more rigid structure, which is an interesting field of study in the aspect of viscoelastic behavior. Whipped cream's quality attributes are mainly affected by the rheological and structural properties. Since measuring the viscoelasticity of whipped cream is difficult, finding a correlation between the rheological properties and microstructure of the cream can be useful, and therefore, the microstructure of cream was investigated by cryo‐scanning electron microcopy methods. Noda & Shiinoki (1986) have studied on this, and they have found a relation between the rheological properties and microstructure of whipped cream (Noda & Shiinoki, 1986). Several factors such as fat content, processing conditions, and stabilizer/emulsifier addition can change the structural properties of whipped cream (Bruhn & Bruhn, 1988). The minimum fat content of cream must be 30% to have a rigid foam structure, and the fat globule size would be in the range of 15–20 μm (Graf & Muller, 1965). In order to promote partial coalescence and improve rigidity to the air bubble interface, at least 40% of the fat should be crystalline (Darling, 1982).

Stability and rigidity of whipped cream are essential parameters in many food industries such as confectionary products. Addition of low molecular weight emulsifiers or stabilizers promotes the adsorption of partially coalesced fat at the air interface through a lowering of interfacial tension (Anderson & Brooker, 1988; Paquin & Dickinson, 1991). However, the viscosity of the aqueous phase can be increased by the addition of large molecule polysaccharide stabilizers. It has been reported that carrageenan can interact with casein micelles and forms glycomacropeptide complex which would afford cohesion between membranes and the serum adding overall structural integrity to the foam. Therefore, several studies have been performed on the cream stabilizers including Aertex cream stabilizer (Smith, Goff, & Kakuda, 2000), locust bean gum and κ‐carrageenan (Camacho, Martínes‐Navarrete, & Chiralt, 2005), sodium caseinate, whey proteins, hydroxypropyl methylcellulose, and xanthan gum on the stability of whipped cream (Zhao, Zhao, Yang, & Cui, 2008, 2009).

Due to the functional and technological properties of whey, it has been found many applications in dairy products such as whipped cream and dairy foams. Indeed, the proteins can stabilize emulsions and foams (Nicorescu et al., 2008; Rullier, Axelos, Langevin, & Novales, 2010). Proteins stabilize foams by strongly adsorbing to the air–water interfaces, forming viscoelastic adsorbed layers, and leading to a protein network with high viscosity (Rullier et al., 2010). Several studies have been performed on the foaming properties and stability of whipped cream by whey proteins (Rullier, Novales, & Axelos, 2008; Zhu & Domodaran, 1994). In comparison with whey, modified whey proteins have indicated more stability of whipped creams (Sajedi, Nasirpour, Keramat, & Desobry, 2014).

Rice bran protein (RBP) has been considered as a suitable plant protein which has unique nutritional characteristics including reasonable protein efficiency ratio (2.0–2.5), high lysine content, more complete amino acid profile, and high digestibility (>90%) in comparison with other whole grain cereals or legumes (Wang et al., 1999; Juliano, 1994). It has also fascinating functional properties including foaming and emulsifying properties, which attracts researchers to get better understanding on their properties. For example, functional properties of rice bran protein isolate at different pH levels have been investigated (Esmaeili, Rafe, Shahidi, & Ghorbani Hasan‐Saraei, 2015). It has found that RBP had higher surface hydrophobicity than that of casein and ovalbumin which can be utilized in the air–water interface systems. Moreover, as pH approaches to alkaline conditions, the protein solubility, and emulsifying and foaming properties of RBP have been improved and resulted in more overrun, which implies its excellent quality in making stable emulsions. In another work, the rheological behavior of RBP has been compared with some hydrocolloids such as xanthan, guar, and locust bean gum (Rafe, Mousavi, & Shahidi, 2014). Results have shown that RBP had a weak network and non‐gelling ability which can be combined with gelling biopolymers such as whey protein. Therefore, the protein–protein interactions between RBP and whey were the issue of another survey to determine the compatibility of binary mixture of RBP and whey (Rafe, Vahedi, & Ghorbani Hasan‐Sarei, 2016). It has been understood the elasticity of binary mixed system of RBP and whey protein concentrate was more than that of single biopolymer. However, the mixed system has shown thermodynamic compatibility and application feasibility in dairy formula and desserts, but adding fibrils of RBP to whey proteins induced more syneresis and less water‐holding capacity. In order to improve the people's health and decrease the cost of product, developing a low‐fat whipped cream is interesting. Therefore, the aim of the current work was to evaluate the rheological and structural properties of whipped cream influenced by adding RBP at varying levels to creams with different fat content to reduce the fat and produce a low‐fat whipped cream.

## 2 MATERIALS AND METHODS

### 2.1 Materials

The commercially dried rough rice of Tarom cultivar was kindly provided by Rice‐lands of Falah, Inc. (Sari, Mazandaran, Iran). All the ingredients were of analytical grade and purchased from Sigma‐Aldrich (St. Louis, MO, USA).

### 2.2 Preparation of RBP

Rough rice (12% moisture content [wb]) was dehulled by a rice husker and debranned by a McGill No. 2 mill for 30 s. Then, the bran was immediately defatted to prevent lipid oxidation. Rice bran protein was extracted as previously described (Rafe et al., 2014). The obtained RBP was freeze‐dried (Freeze dryer FDU‐8624, Operon, Gimpo city, Korea) and stored at −5°C for further experiments. The protein content was measured by the Kjeldahl method, and the other chemical composition of RBP such as crude fiber was determined based on our previous work (Rafe et al., 2014). The content of moisture (925.10), ash (923.03), fiber (920.86), crude fat (920.39), and crude protein (920.87) was determined by the Association of Official Analytical Chemists methods (AOAC, 2002), and the content of carbohydrate was calculated by subtracting the amount of other compounds from 100. The chemical composition of Tarom RBP on the basis of weight of the protein showed it contains protein, fiber, carbohydrate, ash, and moisture as 77.61%, 14.20%, 4.65%, 1.78%, and 3.33%, respectively.

### 2.3 Rheological assay

#### 2.3.1 Flow behavior

Whipped cream with 25% (low‐fat) and 35% (high‐fat) at varying concentrations of RBP from 1% to 3% was used. The samples were conditioned at 10°C for 2 min, and then, the shear rate was increased from 0.1 to 1,000 s^−1^. Then, the experimental flow curve was plotted in shear stress (*τ*) versus shear rate (*γ*). The apparent shear viscosity (*η*_a_) was determined as a function of increasing shear rate in the ramp‐up mode. The rheological behavior of cream with varying amount of RBP was fitted by using different models such as power‐law (Equation (1)), Bingham (Equation (2)), and Casson (Equation (3)) as follows:

(1) $$\tau =K\gamma^{n}$$

(2) $$\tau =\tau_{0}+K\gamma^{n}$$

(3) $$\sqrt{\tau }=\sqrt{\tau_{0}}+\eta \sqrt{\gamma }$$

where *τ*_0_ is the yield stress, *η* is the apparent viscosity, *k* is the consistency index, and *n* is the power‐law index. The correlation coefficient (*R* ^2^) was determined to show the goodness of the model.

#### 2.3.2 Dynamic oscillatory measurements

Small amplitude oscillatory shear (SAOS) measurements of the whipped cream were carried out by a controlled stress/strain rheometer (Paar Physica Rheometer, MCR 301, Anton Paar GmbH, Germany), which was fitted by parallel‐plate geometry (ϕ = 50 mm) with 1 mm gap. The experiments were performed in isotherm conditions, which was controlled fast and precisely by a Peltier system (Viscotherm VT2, Paar Physica) at 10°C and equilibrated at least 10 min before each run. By using the low temperature, the fat congeals on the acrylic plate. Firstly, foam was scooped with a spoon and located at the center of the plate. Then, the top plate was lowered gently by manually resisting the ramp to minimize the damage to the foam structure. Excess whipped cream was removed from the rim of the acrylic plate to decrease the edge effect. The rim of the sample was covered by a thin layer of paraffin to prevent evaporation during the measurement.

Prior to the mechanical tests, strain sweep measurements were performed to determine linear viscoelastic region (LVE), where dynamic G′ and G″ are independent of strain amplitude (Peng, Ren, Zhong, Cao, & Sun, 2010). For comprehensive investigation on the rheological properties of complex coacervate, G′, G″, loss tangent (tan δ), the limiting/critical value of stress (*τ*_c_) at LVE region, the fracture stress and strain, and crossover point were determined. The critical value of stress (*τ*_c_) determined through the intersection of two asymptotic lines drawn through the initial and post‐breakdown modulus data can be considered to be an approximate measure of the yield stress of the material (Pai & Khan, 2002).

Frequency sweep provides suitable knowledge about network structure. Therefore, it was carried out over a range of 0.1–100 Hz to investigate the viscoelastic behavior of cream at 10°C. The degree of frequency dependence of elastic modulus is considered as an indication of the viscoelastic nature of a material. Thus, the degrees of frequency dependence of elastic (Equation (4)) and viscous moduli (Equation (5)) were determined by power‐law model as follows (Khondkar, Tester, Hudson, Karkalas, & Morrow, 2007; Ramkumar, Bhattacharya, Menjivar, & Huang, 1996).

(4) $$G^{\prime }=k\prime \omega^{p}$$

(5) $$G^{\prime \prime }=k^{\prime \prime }\omega^{q}$$

where *k*′ and *k*″ (Pa.s) are intercepts, *p* and *q* are the viscoelastic components of elastic and viscous moduli, and *ω* is oscillatory frequency (Hz).

### 2.4 Microstructure

In order to get micrograph of the whipped cream, the samples were dehydrated in graded acetone solutions at varying levels. Then, they were fixed in the 2.5% glutaraldehyde in cacodylate buffer (pH 7.2) for at least 1 hr. The samples were coated by gold, and 10–15 regions of each sample were subjected to the SEM. The microstructure of the cream was observed under scanning electron microscopy (SEM, model SC 7620, England) with an accelerating voltage of 25 kV and at 3,000 magnification.

### 2.5 Statistical analysis

Whipped cream with 25% (low‐fat) and 35% (high‐fat) at varying concentrations of RBP from 1% to 3% was used. All the rheological and structural analysis was performed at least in triplicates. The obtained results were examined by analysis of variance (ANOVA) at the confidence level of 5%. Rheological data and graphs were analyzed by Rheoplus software (version 3.40 Anton Paar GmbH, Germany) and SigmaPlot software (version 8.0; Jandel Scientific, Corte Madera, CA, USA), respectively.

## 3 RESULTS AND DISCUSSION

### 3.1 Flow behavior

**Figure 1:**
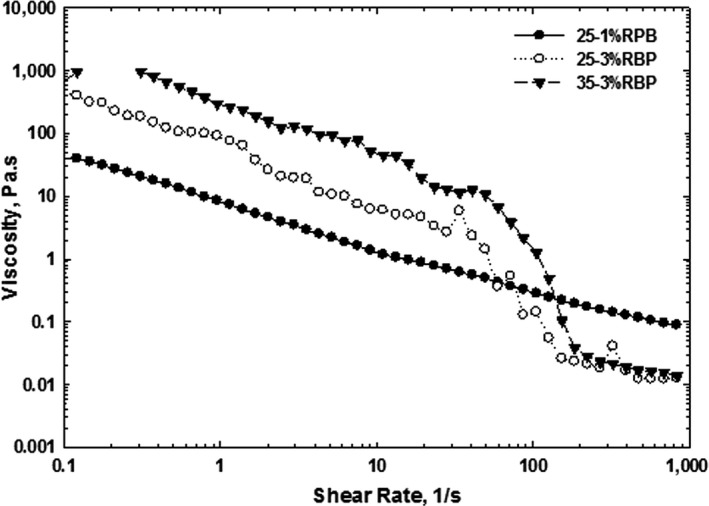
The effect of rice bran protein addition on the apparent viscosity of the whipped cream after 1‐day storage at 4°C

The flow curve of whipped cream with 25% and 30% fat content with varying RBP is displayed in Figure 1. All the samples showed shear rate dependency and shear‐thinning behavior (non‐Newtonian fluid) which is in agreement with previous works on creams (Hussain, Truong, Bansal, & Bhandari, 2016; Walstra & Jenness, 1984). The pseudoplasticity of cream can be attributed to the formation of clusters or aggregates of fat globules, which disrupts at the higher shear rates (Quemada, 1998). The shear forces were strong enough at very low shear rates to disrupt the secondary bonds holding the particles together, resulting in cluster or aggregate deformation or disruption, which finally led to a sharp reduction of the apparent viscosity (Izidoro, Scheer, Sierakowski, & Haminiuk, 2008; Lim, Swanson, Ross, Clark, 2008). Increase in shear rate induced to higher shear forces, facilitating either formation of irregular shapes of aggregates or disruption of aggregates. As a result, the apparent viscosity was decreased with increasing shear rate (Walstra & Jenness, 1984). The effect of the apparent viscosity became less effective when shearing forces surpassed the attractive forces binding fat droplets/globules together. Consequently, the creams revealed low viscosity values at high shear rates as shown in Figure 1. However, cream samples containing 3% RBP showed a rapid reduction in viscosity at shear rates of 50–100 s^−1^. This behavior can be related to the effect of RBP which induce sharp reduction in the apparent viscosity. Although, the high amount of η_a_ was achieved for cream with 35% fat and 3% RBP at the low shear rates. On the other hand, at low shear rates >50 s^−1^, the order of η_a_ was for cream with 35% fat and 3% RBP; cream with 25% fat and 3% RBP and cream with 25% fat and 1% RBP. It can be concluded the effect of fat on the viscosity of cream can be compensated by adding RBP.

In order to better explain the rheological behavior, different models such as power‐law, Casson, and Bingham models were evaluated (Table 1). According to the results, among the different models, the best fitting with high correlation coefficient (*R* ^2^ was between 0.97 and 0.99) was obtained for the power‐law model. Since the *n* value of the samples was <1 at all conditions, the foam exhibited a pseudoplastic behavior. In the current work, it was found that *n* varied from 0.29 to 0.42. The lowest shear‐thinning index (*n* = 0.294) was obtained for the cream sample with 25% fat and 1% RBP which can be attributed to the RBP and its less interaction with other particles. However, the cream sample containing 35% fat and 3% RBP had also shown the low index (*n* = 0.298) which confirmed the firmer structure of cream. In contrast, the highest *n* value was achieved for cream with 25% fat and 3% RBP (*n* = 0.42). It may be attributed to the RBP molecular structure which is a protein with many branched structure that prevents from filling the void spaces among the foam. The maximum *K*, consistency index, obtained was 9.41 for the cream with 35% fat and 3% RBP which approved its strong foam structure. In contrary, the low consistency index was acquired for the cream with 25% fat and 3% RBP (4.33). In terms of consistency index (*K*), the samples with higher fat content and protein showed high consistency, which might be explained by the structural built‐up or gel formation developed under high protein content of cream (Velez‐Ruiz & Barbosa‐Canovas, 1998).

**Table 1:** Correlation coefficient of the different models was used for the different formula of the whipping cream

Whipped cream formula	Correlation coefficient of models (*R* ^2^)^a^		
	Power‐law	Bingham	Casson
25%–1%RBP	0.98	0.85	0.84
25%–3%RBP	0.99	0.86	0.86
35%–3%RBP	0.97	0.87	0.87

a All the experiment in the flow behavior was carried out in triplicates and *p* < 0.05.

The minimum shear stress required to initialize the flow can be defined as yield stress which is a critical quality control parameter in industrial processing, transportation, or storage. As the more fat content in the whipped cream, the more yield stress (*τ*_0_) values were achieved, which illustrated the change tendency of apparent viscosity. Therefore, the highest *τ*_0_ was obtained for cream with 35% fat and 3% RBP (5.0 Pa), and the lowest *τ*_0_ was 1.49 Pa for the cream with 25% fat and 3% RBP. It was also indicated the more stable emulsions, due to the feasibility of structural change from flow resulting in instability, were reduced (Innocente, Biasutti, Venir, Spaziani, & Marchesini, 2009). Overall, the yield stress value can be increased by increasing the particle volume fraction, interparticle forces, and decreasing particle sizes (Genovese, Lozano, & Rao, 2007).

### 3.2 Mechanical properties

**Figure 2:**
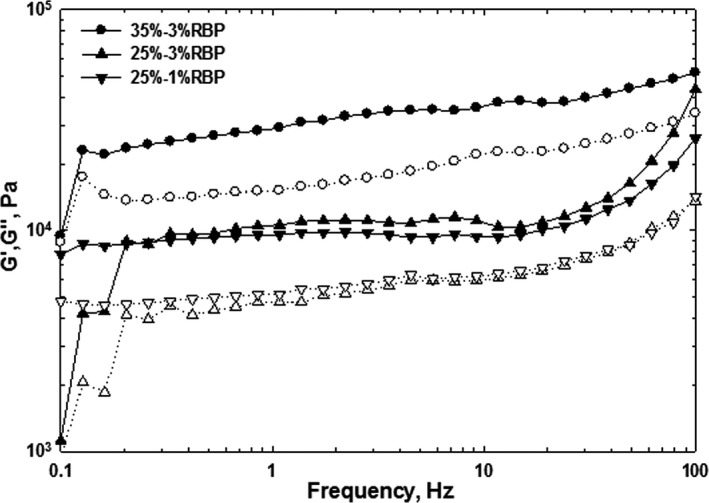
Effect of rice bran protein addition on the storage modulus (G′, filled symbols) and loss modulus (G″, blank symbols) of whipping cream

Dynamic oscillatory testing designed to measure structure without deformation was the method chosen to measure the fragile foam structure. The viscoelastic properties of the whipped cream were subjected to a frequency sweep test of elastic modulus (G′) and viscous modulus (G″) after storing the samples at 4°C for 1 day. All samples showed frequency dependency (Figure 2), and by increasing the frequency, both moduli were increased. For all the samples, the G′ value was more than G″ revealing a greater contribution of elastic properties and consequently demonstrating solid‐like systems which exhibit limited transitional movement upon shear stress due to the fat crystal complex. The G″ values of the samples produced similar values and trends of generally increasing and then plateauing, whereas G′ values obtained for each sample were different from other samples. The highest value of G′ was obtained for the cream sample of 35% fat and 3% RBP. The results showed that by increasing fat and RBP, the elasticity of the cream was improved and it is feasible to enhance the foam structure. According to the report of Speroni et al. (2009), these rheological characteristics indicated weak gel systems, in which the protein segments were adsorbed at oil–water interface and formed pseudo‐gel network. The sample submitted to a higher homogenization pressure showed an increase in G′ and G″, as well as a larger gap between G′ and G″.

**Figure 3:**
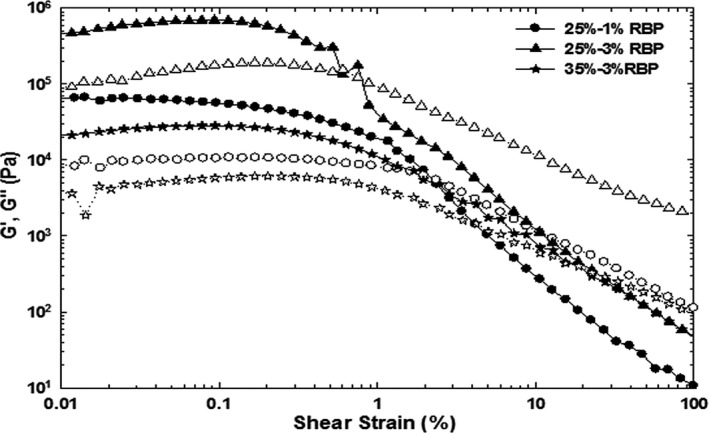
The effect of rice bran protein addition on the storage modulus (G′, filled symbols) and loss modulus (G″, blank symbols) of whipping cream after 1‐day storage at 4°C

The phase angle (*δ*) refers to the inverse tangent of the ratio of G″ to G′. δ gives an indication of a samples rheological characteristics, with a phase angle 0° and 90° demonstrating behavior of a purely elastic solid and viscous fluid, respectively (Weiss & McClements, 2009). Material exhibits viscoelastic behavior if the phase angle is between 0° and 90° (Steffe, 1996). A small value of the phase angle (i.e., 20°) shows pronounced elastic (gel) behavior of a material (a large value of G′ in comparison with G″). Figure 3 illustrates the transition of a gel‐like form due to the electrostatic repulsion between the fat globules in close proximity, to a liquid‐like form with increasing globular size achieving a phase angle near to 45° for cream sample with 25% fat and 1% RBP, indicating behavior of a viscous fluid.

The responses of whipped cream to treatment differences were compared with evaluating the dependency of log G′ on log frequency (Hz). Regression lines for slope comparison showed the distortion following loading of the sample and the inertia at low‐frequency levels, although the data points were kept to improve statistical contrasts.

### 3.3 Microstructure of cream with RBP

**Figure 4:**
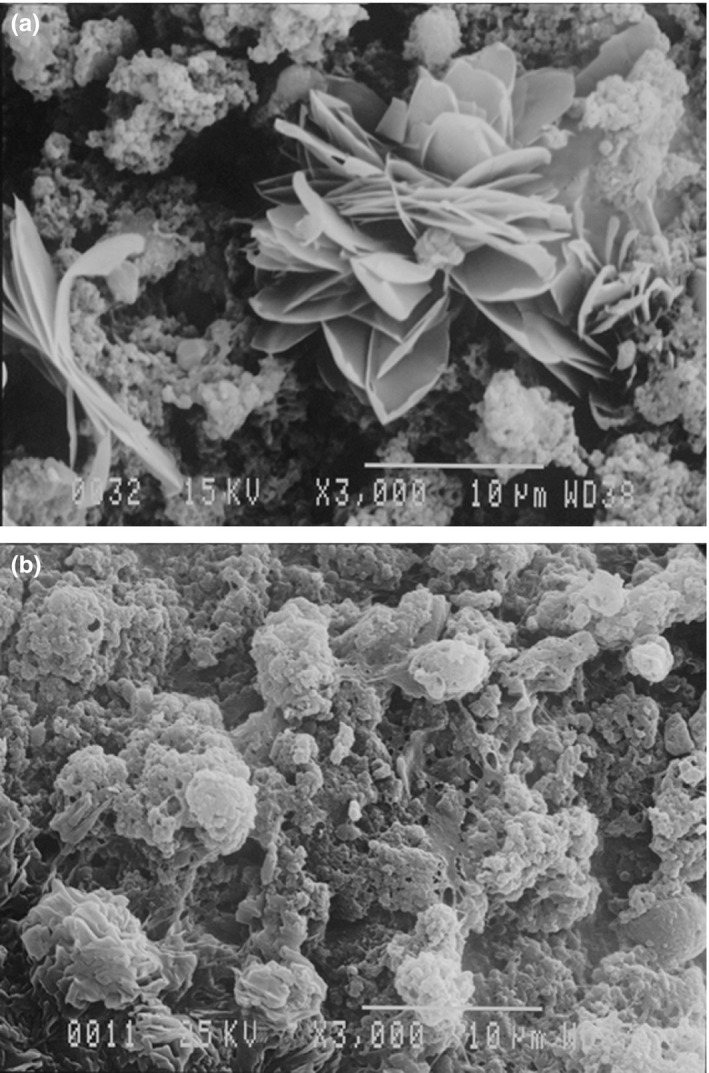
SEM micrographs of whipped cream containing 3% RBP at (a) 25% fat and (b) 35% fat (Magnification 3,000, voltage 25 kv)

Micrograph of the whipped creams mixed with varying levels of RBP was examined by SEM to elucidate the detail information on the cream formation and structure (Figure 4). The micrographs indicated that the cream structure has relatively large globular aggregates in a network and develops large pores, which let to retain sufficient water/air (Figure 4a). Therefore, it can be stated that whipped cream developed a continuous structure which can be found in the other food structures (Van den Berg et al., 2007). It has been stated that RBP had more fibril structure and has a lentil‐like structure (Rafe et al., 2014). By increasing the fat content of cream from 25% to 35%, the voids and spaces in the cream were significantly decreased and the pores become less which improve the foam structure. Therefore, it can be concluded the cream with more fat have the more overrun and stability. However, the RBP has also improved its stability and overrun, and the amorphous structure has been observed for fibrillar aggregates of RBP, which is affected by concentration (Zhang and Huang 2014). Similar structure of the other protein mixed systems such as gellan gels and mixed systems like WPI‐κ‐carrageenan and basil seed gum‐β‐lactogolbulin has been determined (Van den Berg and others 2007; Ould Eleya and Turgeon 2000). It is necessary to mention that gels containing high porosity, such as bicontinuous and coarse stranded gels, release the highest amount of serum. Indeed, by RBP addition to whipped cream, a coral‐like structure may be developed, which reveals gels with fibrils tended to show syneresis. They have declared that the gels with fibrils had a lower water‐holding capacity (Zhang and Huang, 2014).

## 4 CONCLUSION

Rheological and microstructure of whipped cream with 25% and 30% fat content with varying RBP have been investigated here. The pseudoplasticity behavior of cream could be attributed to the formation of clusters or aggregates of fat globules, which disrupts at the higher shear rates. However, cream samples containing 3% RBP showed a rapid reduction in viscosity at shear rates of 50–100 s^−1^. This behavior can be related to the effect of RBP which induce sharp reduction in the apparent viscosity. Although, the high amount of *η*_a_ was achieved for cream with 35% fat and 3% RBP at the low shear rates. Among the different used models, the best fitting with high correlation coefficient was obtained for the power‐law model. Since the *n* value of the samples was less than 1 at all conditions, the foam exhibited a pseudoplastic behavior. It may be attributed to the RBP molecular structure which is a protein with many branched structure that prevents from filling the void spaces among the foam. The maximum *K*, consistency index, obtained was 9.41 for the cream with 35% fat and 3% RBP which approved its strong foam structure. The highest *τ*_0_ was obtained for cream with 35% fat and 3% RBP (5.0 Pa), and the lowest *τ*_0_ was 1.49 Pa for the cream with 25% fat and 3% RBP. The highest value of G′ was obtained for the cream sample of 35% fat and 3% RBP. The results showed that by increasing fat and RBP, the elasticity of the cream was improved and it is feasible to enhance the foam structure. The micrographs showed that the cream structure has relatively large globular aggregates in a network and develops large pores, which permit to retain sufficient water/air. By increasing the fat content of cream from 25% to 35%, the voids and spaces in the cream were significantly decreased and the pores become less which improve the foam structure. Therefore, it can be concluded the cream with more fat have the more overrun and stability. In general, it is possible to improve the foam structure of cream by substituting fat by RBP.

## CONFLICT OF INTEREST

The authors declare that they have no conflict of interest.

## ETHICAL STATEMENT

The study does not involve any human or animal testing.
